# Prevalence and immediate outcomes of low birth weight neonates born of pre-eclamptic women at Moi Teaching and Referral Hospital, Kenya

**DOI:** 10.11604/pamj.2023.44.31.37975

**Published:** 2023-01-17

**Authors:** Lina Sigei, Emily Muthoni Nyaga, Benson Milimo

**Affiliations:** 1 School of Nursing and Midwifery, Moi University, Eldoret, Kenya

**Keywords:** Low birth weight, preeclampsia, immediate birth outcomes

## Abstract

**Introduction:**

pre-eclampsia has been linked to poor neonatal outcomes such as; stillbirth, low birth weight (LBW), prematurity and neonatal morbidities owing to utero-placental insufficiency. The study objective was to determine the prevalence of LBW and immediate (within 24 hours) birth outcomes of LBW neonates born to pre-eclamptic women at Moi Teaching and Referral Hospital (MTRH), Kenya.

**Methods:**

a descriptive cross-sectional study was conducted among 364 participants (346 singletons and 9 twins) born to pre-eclamptic women at MTRH. A study tool was used to gather data on birth weight and neonatal outcomes. Data was cleaned, coded and entered into SPSS version 22 for analysis. Descriptive statistics were computed for the prevalence of LBW and immediate neonatal outcomes.

**Results:**

the study found an LBW prevalence of 180(49.5%) and prematurity of 81(45%). Of the LBW neonates (n=180), 162(90%) were alive and 18(10%) were stillbirths. Their immediate morbidities were, birth asphyxia 51(28.7%), neonatal jaundice 38(21%), hypothermia 18(7.9%) and neonatal sepsis 1(0.7%). Of the neonates that were born alive; 107(59.2%) were admitted to level II nursery care, 53(29.5%) were rooming in with their mothers and 20(11.3%) died within 24 hours.

**Conclusion:**

preeclampsia contributes to LBW, nursery admissions and morbidity/mortality of neonates necessitating the need for neonatal units' preparedness for prompt and appropriate management to avert death.

## Introduction

Birth weight is the first weight recorded after birth, ideally measured within the first hours after birth, prior to the significant weight loss that occurs after birth. Low birth weight (LBW) is a birth weight of less than 2500 grams (up to and including 2499grams) and is further categorized into very low birth weight (VLBW, <1500grams) and extremely low birth weight (ELBW, <1000grams) [1]. The LBW is a crucial indicator of child´s vulnerability to mortality, morbidity, delayed growth and development, chronic diseases later in life, and chances of survival [2,3]. The increased neonatal mortality among LBW neonates has been attributed to susceptibility to hypoglycemia, hypothermia, birth asphyxia, trauma, respiratory disorders, and neonatal sepsis [4]. For instance, Tshehla *et al*. (2019) [3] reported a mortality rate twenty times higher among LBW compared to normal birth weight neonates. Notably, the global prevalence of LBW in 2015 stood at 15.5% (20.5 million neonates) with the majority (91%) arising from low and middle-income countries, 24% from sub-Saharan Africa, and 11% from Kenya [5,6].

Preeclampsia is a known part of hypertensive disorders of pregnancy and is classically defined as hypertension and proteinuria or hypertension with end-organ damage after 20 weeks of gestation. Majorly presents with visual disturbances, headaches, swelling, excessive weight gain, and abdominal pain. Preeclampsia remains a common health problem with a global incidence of between 2 to 10%. Of concern, the health problem often goes under-recognized and undertreated impacting greatly on birth weight [7-9]. Owing to acute or chronic uteroplacental insufficiency, preeclampsia has been associated with intrauterine growth restriction, LBW, prematurity, stillbirth, admission to neonatal intensive care unit, and perinatal death [10-12]. A systematic review and meta-analysis conducted in Ethiopia reported an LBW prevalence of 39.7% among neonates born to pre-eclamptic women [13]. In addition, a retrospective study in Jamaica by McKenzie *et al*. (2019) [14] reported pre-eclamptic women as more likely to give birth to LBW (OR = 2.8; CI: 2.2 - 3.5), small for gestational age (OR = 2.3; CI: 1.9-2.9) or preterm neonates (OR = 2.5; CO: 2.0-3.0) than normotensive women.

Although Moi Teaching and Referral Hospital (MTRH), Kenya, has had a lower prevalence of LBW neonates than the national figure of 22% [15], the figures have been rising for the past three years from 2018 with a prevalence of 8.9%, 10.7% and 11.6% (hospital statistics, 2021). The cost implication to the hospital, families, and society in the care of LBW neonates remains a burden, especially in developing countries. This study's objective was to determine the prevalence and immediate (within 24 hours) outcomes of LBW neonates born to pre-eclamptic women at MTRH, Kenya.

## Methods

**Study design and setting:** a descriptive cross-sectional study was conducted at MTRH, the second largest referral hospital in Kenya located in Uasin Gishu County.

**Sample size:** a sample size of 355 was arrived at using the formula for estimating single population proportion described by Lwanga *et al*. (1990) [16] and a prevalence of LBW of 36.2% from a similar study conducted in Ethiopia by Legesse *et al*. 2019, [17].

**Population:** the target population was all neonates born of women diagnosed with preeclampsia at MTRH during the study period. Approximately 310 neonates are born to pre-eclamptic women every month. The study targeted a study population of 1550 for the five months of data collection.

**Sampling procedure:** four trained research assistants (nursing staff on off-duty working at MTRH) recruited study participants. Women diagnosed with preeclampsia and birthing at the labor ward or maternity theatre had their neonates recruited into the study after delivery. Systematic sampling was employed where every 4^th^ neonate (target population of 1550/sample size of 355) was recruited into the study until the desired sample size was reached. A total of 364 participants (346 singletons and 9 twins) were recruited into the study between 21^st^ March and 20^th^ August 2021.

**Eligibility criteria:** neonates were included in the study if they were; a) born to pre-eclamptic women during the study period, b) delivered within the last 24 hours, and c) delivered at MTRH. Neonates born of women diagnosed with eclampsia were excluded from the study due to the need to terminate the pregnancy.

**Data collection procedures:** a study tool developed by the first author (Lina Sigei) following a thorough review of related literature was reviewed for content by four experts in the field of maternal and neonatal health resulting in the editing of two items in the study tool. In addition, a pilot study was conducted at MTRH among 30 participants a month prior to data collection occasioning a rephrasing of one item and content revision of two items. A Cronbach´s alpha coefficient of 0.7 was obtained for the pre-test population making the tool sufficient for reliability. Participants in the pilot study were excluded from the main study. The study tool obtained data on BWT, gestational age at birth, gender, mode of delivery and neonatal appearance, pulse, grimace, activity, and respirations (APGAR) score. Also obtained were immediate outcomes (state of the neonate at birth including medical diagnosis all obtained from neonates file) such as congenital malformation, need for resuscitation, and morbidities for LBW neonates.

**Statistical analysis:** data collected were coded and entered into Statistical Package for the Social Sciences (SPSS) version 22 database for analysis. The analysis aimed at determining; a) the prevalence of LBW and b) neonatal social demographic factors and immediate birth outcomes of the LBW neonates. Descriptive statistics were computed for neonatal social demographic factors, the prevalence of LBW, and immediate birth outcomes. The results were presented in tables.

**Ethics approval and consent to participate:** the study was approved by the Institutional Research and Ethics Committee (IREC) of Moi Teaching and Referral Hospital; approval number 0003815. In addition, written informed consent was obtained from the mothers prior to data collection. The authors declare that all methods were carried out in accordance with relevant guidelines and regulations for research.

## Results

The findings of the 364 participants revealed an LBW prevalence of 49.5%. An analysis of the LBW participants (n = 180) found that 81(45%) were preterm, 102(56.7%) were males, 18(10%) were twins and 107(59.44%) were delivered via caesarean section. The mean BWT was 2017 (± 417.539) grams, and APGAR scores were 6.4 (± 2.8), 7.2 (±3.1) and 7.7 (±3.1) at one, five and ten minutes respectively (Table 1).

**Table 1:** neonatal characteristics (n=180)

Variable	Category	Frequency	Percentage
Gestation at birth	Completed 37 weeks gestation	99	55.00
	Less than 37 weeks gestation (preterm)	81	45.00
Sex	Male	102	56.67
	Female	78	43.33
Child is twin	Yes	18	10.00
	No	162	90.00
Mode of delivery	Caesarean section	107	59.44
	Normal	73	40.56
**Variable**	**Category**	**Mean**	**Standard deviation**
Birth weight		2017.01	417.539
APGAR score	At 1 Minute	6.44	2.773
	At 5 Minute	7.23	3.089
	At 10 Minute	7.66	3.123

An analysis of the immediate outcome of the LBW neonates found that at birth 162(90%) participants were alive, 17(9.4%) were fresh still births while 1(0. 6%) was macerated still birth. Notably, 12(7%) participants had congenital anomalies while 67(37%) were resuscitated after birth. The neonatal morbidities found were; 51(28.7%) birth asphyxia, 38(21%) neonatal jaundice, 18(7.9%) hypothermia, 3(1.7%) multiple morbidities and 1(0.7%) neonatal sepsis. At the end of 24 hours following delivery, the study found that 107(59.2%) of the participants were admitted at special care nursery, 53(29.5%) were rooming in with their mothers and 20(11.3%) had died (Table 2).

**Table 2:** immediate outcomes of the low birth weight neonates (n=180)

Variable	Category	Frequency	Percentage
Birth outcome	Born alive	162	90.00
	Fresh still birth	17	9.44
	Macerated still birth	1	0.56
Congenital malformation	No	168	93.00
	Yes	12	07.00
Neonatal resuscitation	No	167	63.00
	Yes	13	37.00
Neonatal morbidities	Birth asphyxia	51	28.73
	Neonatal jaundice	38	21.00
	Hypothermia	18	07.90
	Neonatal sepsis	1	0.68
	Multiple morbidities	3	1.70
	No morbidities	69	40.00
Neonate state at the end of 24 hours after birth	Alive with mother	53	29.53
	Admitted to nursery	107	59.18
	Died	20	11.29

## Discussion

The study findings revealed an LBW prevalence of 49. Five percent (5%) similar to findings reported in Nigeria by Yilgwan *et al*. (2020) [18], Ethiopia by Legesse *et al*. (2019) [17], and Brazil by Anselmini *et al*. (2018) [19] found a prevalence of 42.2%, 36.2%, and 32.7% respectively. Ours though is on the higher side underscoring the importance of measures to mitigate this. Similar to the current study, McKenzie *et al*. (2019) [14] reported a significantly lower mean BTW (2.2±0.9 kilograms) among neonates born to pre-eclamptic women compared to neonates born to normotensive women (2.2±0.9 kilograms). The low birth weight among pre-eclamptic women has been attributed to fetal under-nutrition as a result of uteroplacental vascular insufficiency [20]. Congruent to our study findings, a study in Brazil by Anselmini *et al*. (2018) [19] found 46.2% of neonates were born prematurely by pre-eclamptic women while contrast findings from South Africa by Nathan *et al*. (2018) [21] and Ethiopia by Belay *et al*. (2020) [22] reported prematurity of 70% and 19.5% respectively. That about two-thirds of the LBW neonates were delivered via caesarean section owing to non-reassuring fetal status and worsening preeclampsia or impending eclampsia was congruent with other studies that have reported caesarian section as the most common mode of delivery among pre-eclamptic women in the quest to mitigate adverse perinatal outcomes [23-25].

The mean APGAR scores for the participants were consistent with other studies that have reported low APGAR scores in LBW neonates at one and five minutes compared to normal weight neonates [AOR = 0.52 (95%CI: 0.37-0.73)] [26-28]. Moreover, 10% of the LBW participants in the current study were stillbirths comparable to findings of 17.7% in South Africa [21], 8.9% in Ethiopia [13], and 6.7% in Brazil [19]. Preeclampsia has been reported to pose a significant risk for intrauterine fetal demise due to placental insufficiency contributing to 2.1% of stillbirths [29]. In addition, our study found that 37% of the participants were resuscitated after birth similar to the findings of 42.7% in Thailand by Kongwattanakul *et al*. (2018) [30].

Congruent to our study findings, other studies have reported birth asphyxia as the leading cause of neonatal morbidity among neonates born to pre-eclamptic women owing to lung immaturity and insufficient surfactant production [23,25,31]. In contrast, some studies have reported preeclampsia as being protective of respiratory morbidity by accelerating fetal lung maturation [32-34]. The study also found that neonatal jaundice occurred in 21% of the participants similar to findings in Iran by Boskabadi *et al*. (2020) [35] that reported a prevalence of 30.9%. Similarly, other studies have reported an increased risk of developing jaundice among LBW born to pre-eclamptic women [36,37]. Our study findings on the state of the participants at the end of 24 hours after birth are congruent with a study by McKenzie *et al*.(2019) [14] that established that 60% of the LBW neonates born to pre-eclamptic women were admitted to the neonatal unit, 24.2% were rooming in with their mothers and 15.8% had died.

**Limitations:** this study was carried out at a national referral hospital that had a possible bias of admitting high-risk pregnancies referred from other regions in the country hence the findings may not be generalizable. In addition, the study included twins who may not share the same risk factors as the singletons.

## Conclusion

This study was carried out at a national referral hospital that had a possible bias of admitting high-risk pregnancies referred from other regions in the country hence the findings may not be generalizable. In addition, the study included twins who may not share the same risk factors as the singletons. Preeclampsia contributes to LBW, nursery admissions, and morbidity/mortality of neonates necessitating the need for neonatal units’ preparedness for prompt and appropriate management to avert death.

### What is known about this topic

- *Preeclampsia contributes to stillbirth, low birth weight, prematurity, and morbidity due to uteroplacental insufficiency*.

### What this study adds

- *The findings of low birth weight (49%), prematurity (45%), and fresh stillbirth (9%) among pre-eclamptic women at MTRH, Kenya*.
